# Utilization of Exception Requests After Implementation of the National Heart Review Board for Pediatrics

**DOI:** 10.1111/petr.70204

**Published:** 2025-10-07

**Authors:** Rachel E. Harris, David W. Bearl, Lydia K. Wright

**Affiliations:** ^1^ Monroe Carell Jr. Children's Hospital at Vanderbilt Nashville Tennessee USA; ^2^ Nationwide Children's Hospital Columbus Ohio USA

**Keywords:** cardiomyopathy, exceptions, single ventricle congenital heart disease, vasculopathy

## Abstract

**Background:**

In 2020, the Organ Procurement and Transplantation Network authorized the creation of a National Heart Review Board for Pediatrics along with a guidance document. The purpose of this study was to evaluate the utilization of exception requests and approval rates after the implementation of the new review board.

**Methods:**

This was a retrospective review of 1A and 1B exception requests between June 2021 and January 2023. Exception request narratives were reviewed to determine if the request followed guidance and then analyzed the frequency of those approved by diagnosis.

**Results:**

There were 591 exception requests submitted for 419 candidates with an overall approval rate of 91%. Only 55% of the exceptions followed the guidance, but of those, 97% were approved. Exceptions submitted that did not follow the guidance had a lower approval rate of 85%. 1A exception requests that followed the guidance were significantly more likely to be approved than those that did not (97% vs. 62%, respectively, *p* < 0.05). For 1B requests, approval rates did not differ between requests that followed the guidance and those that did not (98% vs. 95%, respectively, *p* = 0.15). Candidates with single ventricle congenital heart disease made up 59% of 1B exception requests. There is no 1B exception guidance for candidates with dilated or hypertrophic cardiomyopathy; however, requests were still submitted and 95% were approved.

**Conclusion:**

Most status 1A exceptions being granted followed the guidance document from OPTN. While the greatest number of requests for 1B exceptions were for single ventricle physiology candidates, many exceptions outside the guidance are being sought and granted.

## Graphical abstract

The overall approval rate of exception requests was 91%. Most status 1A exceptions being granted followed guidelines, which address four underlying diagnosis categories. While the greatest number of requests for 1B exceptions were for single ventricle physiology candidates, many exceptions outside the guidance document are sought and granted.

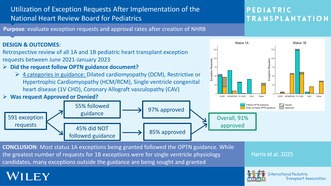

**Abbreviations**
1V CHD: single ventricle congenital heart disease
CAV: cardiac allograft vasculopathy
DCM: dilated cardiomyopathy
HCM/RCM: hypertrophic or restrictive cardiomyopathy
NHRB: National Heart Review Board for Pediatrics
OPTN: Organ Procurement and Transplantation Network

## 1 Introduction

The Organ Procurement and Transplantation Network (OPTN) allows exceptions to standard listing criteria for both adult and pediatric heart transplant candidates in the United States. The goal of status exceptions is to make access to transplant more equitable among patients with similar levels of illness and medical urgency.

The use of status exceptions for pediatric heart transplant candidates has varied with changes in organ allocation policy. Prior to the 2016 change in pediatric heart allocation, there was found to be no difference in waitlist mortality between candidates listed 1A by exception compared to those listed by standard criteria [1]. However, several studies have found an overall increase in the number of listings using exception criteria since the implementation of the revised listing criteria with lower mortality in candidates listed with exceptions [2, 3]. Studies have also demonstrated significant center and regional variability in the use of status exceptions for pediatric patients listed for heart transplantation under the current allocation system, likely exacerbated by regional review boards comprised mostly of adult providers without expertise in pediatric or congenital heart transplant [2, 4].

Recognizing the above challenges, in 2020 the OPTN authorized the creation of a National Heart Review Board (NHRB) for Pediatrics along with additional guidance on what might be considered appropriate status exception requests [5]. The review board consists of experts in the field of pediatric heart transplant, and the purpose of the document is to provide guidance to reduce variability, given that the standard criteria are a less detailed policy than prior iterations. This guidance document addressed four diagnosis categories: dilated cardiomyopathy (DCM), hypertrophic or restrictive cardiomyopathy (HCM/RCM), single ventricle congenital heart disease (1V CHD), and cardiac allograft vasculopathy (CAV) [5]. A summary of the guidance document is shown in Table 1.

**TABLE 1:** Summary of exception request guidance adapted from OPTN Guidance for Pediatric Heart Exception Requests [5].

Category	Candidate may be eligible for status 1A exception	Candidate may be eligible for status 1B exception
Dilated cardiomyopathy (DCM)	Admitted to the transplant hospital and Less than 5 kg on high dose inotropes Less than 10 kg on high dose inotropes with evidence of poor systemic perfusion Mechanical circulatory support is contraindicated	
Restrictive or hypertrophic cardiomyopathy (HCM/RCM)	Admitted to the transplant hospital and On high dose inotropes Had an episode of sudden cardiac death or hemodynamically significant arrhythmia not controlled by medical therapy Had syncopal episodes related to restricted ventricular filling Evidence of increased pulmonary vascular resistance (exceeding 6 WU/m^2^)	
Single ventricle congenital heart disease (1V CHD)	Admitted to the transplant hospital and is experiencing complications related to their 1V CHD (including but not limited to protein‐losing enteropathy, plastic bronchitis, or Fontan circuit thrombosis), and is actively receiving therapy for said complication, without regard for change in the candidate's cardiac support	Palliated through a Fontan procedure, listed for heart transplantation, and has ongoing complications of the Fontan (including, but not limited to protein‐losing enteropathy, plastic bronchitis, or Fontan circuit thrombosis) and is actively receiving therapy for said complication but does not require hospital admission.
Coronary allograft vasculopathy (CAV) and retransplantation	A history of recent cardiac arrest, or signs or symptoms placing patients at high‐risk for sudden cardiac death, including any of the following: A diagnosis of severe CAV similar to ISHLT CAV 3 Significant restrictive hemodynamics Nonsustained ventricular tachycardia Unexplained syncope Inotrope dependence	A history of revascularization (either surgical or transcatheter) for CAV

The purpose of this study was to evaluate exception requests and the approval rates after the implementation of the NHRB for pediatric heart transplant listing.

## 2 Methods

This was a retrospective review of 1A and 1B exception requests and appeals from June 2021, the time that the pediatric NHRB was implemented, through January 2023. This study used data from the Organ Procurement and Transplantation Network (OPTN). The OPTN data system included data on all donor, wait‐listed candidates, and transplant recipients in the US, submitted by the members of the Organ Procurement and Transplantation Network (OPTN). The Health Resources and Services Administration, U.S. Department of Health and Human Services provides oversight to the activities of the OPTN contractor. All data requested from OPTN was approved by HRSA through a data use agreement with Vanderbilt University Medical Center. Exception request narratives were reviewed strictly and evaluated to determine if the request followed OPTN guidance. We then analyzed the frequency of those approved and denied by diagnosis category. Candidates with a diagnosis outside the scope of the guidance document were considered to not be following the guidance document and were still included in the overall analysis.

Descriptive statistics were performed to determine the median and interquartile values for baseline characteristics for candidates for which exception requests were sought. Wilcoxon rank‐sum tests were performed to compare 1A and 1B exception request candidates. A *p* value < 0.05 was considered significant.

## 3 Results

During the study period, 1108 pediatric patients were listed for heart transplantation, of which 338 (35%) were listed by exception. There were 591 exception requests submitted for 419 patients from 55 centers in the United States. Baseline demographics for pediatric heart transplant candidates for which 1A and 1B exception requests were sought are shown in Table 2. There was a total of 276 1A exception requests submitted and 315 1B exception requests submitted. 1A exception request candidates were younger, lower weight, and more likely to be female. Infants less than 1 year old composed 29% of 1A exception requests. 1A requests were more likely to be submitted for candidates with DCM or HCM/RCM, and 1B requests were more likely to be requested for candidates with 1 V CHD.

**TABLE 2:** Baseline demographics for all candidates for which 1A and 1B requests were submitted between June 2021 and January 2023.

	1A requests	1B requests	*p*
	*N* (%), median (IQR)	*N* (%), median (IQR)	
Age at listing (years)	7 (0, 13)	11 (5, 14)	< 0.05
< 1	80 (29%)	20 (6%)	
1–18 years	196 (71%)	295 (94%)	
Age at initial request (years)	7.5 (0, 14)	11 (6, 14)	< 0.05
< 1	72 (26%)	12 (4%)	
1–17	199 (72%)	294 (93%)	
18+	5 (2%)	10 (3%)	
Weight at listing (kg)	21.3 (6.7, 49.5)	35.8 (17.7, 54.0)	< 0.05
Gender			
Male	124 (45%)	174 (55%)	< 0.05
Female	152 (55%)	141 (45%)	
Race			
White	143 (52%)	193 (61%)	< 0.05
Black	51 (19%)	33 (10%)	
Hispanic	59 (21%)	78 (25%)	
Other	23 (8%)	11 (4%)	
Blood type			
A	76 (28%)	98 (31%)	0.39
B	26 (9%)	28 (9%)	
AB	4 (1%)	1 (< 1%)	
O	170 (62%)	188 (60%)	
Diagnosis			
DCM	97 (35%)	10 (3%)	< 0.05
HCM/RCM	74 (27%)	57 (18%)	
1 V CHD	34 (12%)	186 (59%)	
CAV	41 (15%)	25 (8%)	
Other	30 (11%)	37 (12)	
Multiorgan transplant	2 (1%)	3 (1%)	

Overall, there was a 91% approval rate of all exception requests. However, only 55% (326/591) of the exception requests submitted for candidates followed the guidance, but of those, 97% (317/326) were approved. The approval rate for exception requests which did not follow the guidance was only 85% (220/265). Only 12 of the 591 submitted exception requests during the study period were appeals, 9 of which were 1A and 3 were 1B, and only 4/12 (33%) of these were approved.

**FIGURE 1:**
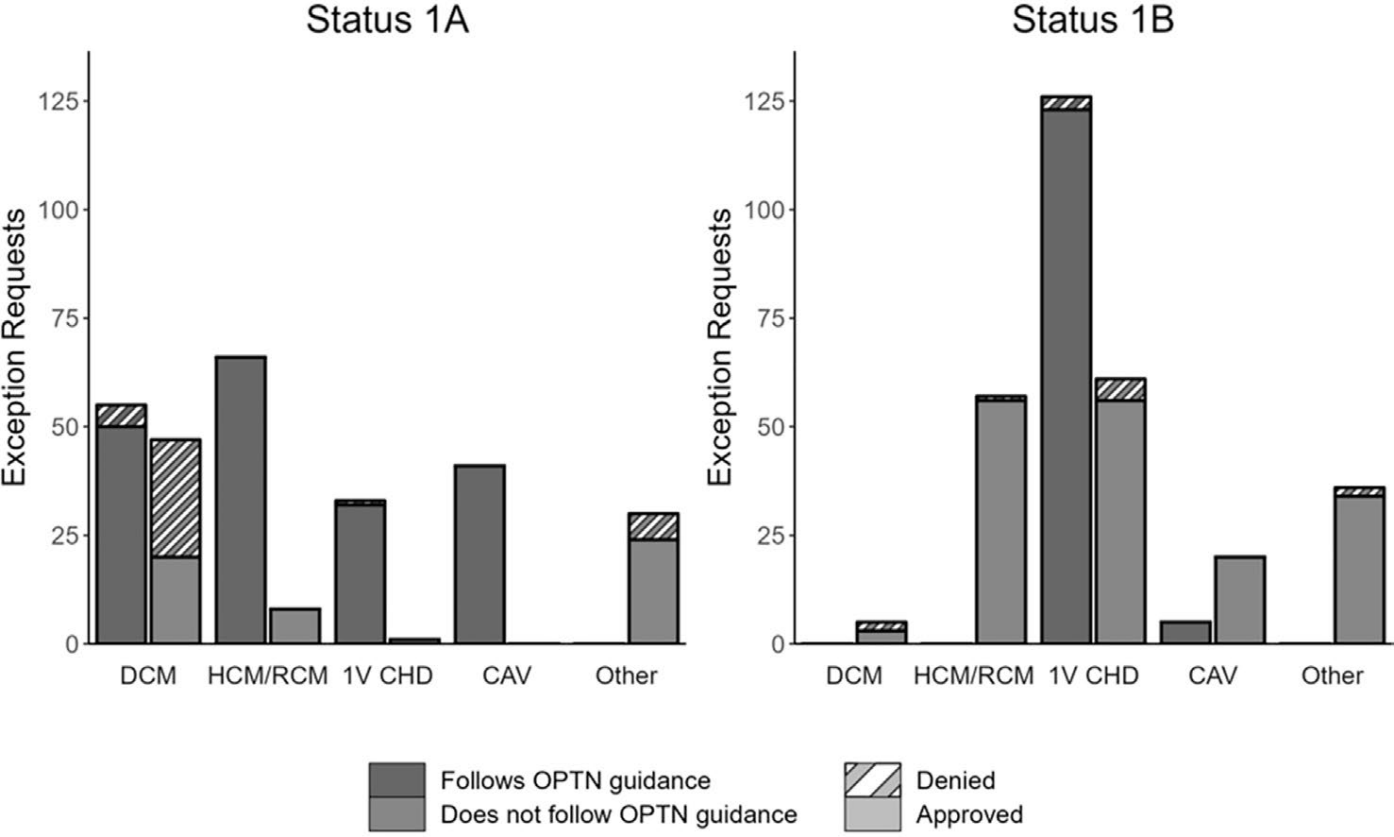
1A and 1B exception requests by diagnosis category broken down by requests that followed the OPTN guidance and requests that did not follow guidance and those which were approved and denied.

Figure 1 demonstrates 1A and 1B exception requests by diagnosis category and requests that followed and did not follow guidance, as well as the proportion which were approved and denied. For 1A requests, exception requests submitted that followed the guidance were significantly more likely to be approved than those that did not (189/195 (97%) vs. 53/86 (62%), *p* < 0.05). For 1B requests, approval rates did not differ between exception requests that followed the guidance and those that did not (128/131 (98%) vs. 169/179 (95%), respectively, *p* = 0.15). None of the exception requests for candidates with DCM or HCM/RCM followed the guidance because there is no guidance for 1B in these categories. However, 1B requests were still submitted and 95% were approved. Candidates with failing Fontan physiology were the majority of 1B exception requests for 1 V CHD category. However, nearly one third of exception requests were for candidates with single ventricle physiology that were not Fontan candidates and 92% were approved.

Roughly 11% of exception requests (50/419 candidates) did not fall into one of the four categories addressed in the guidance document but still had a high overall approval rate of 87%. In this group, there were 21/26 (81%) 1A exception requests approved and 23/24 (96%) 1B exception requests approved. Examples of requests submitted outside of the four categories included biventricular congenital heart disease with dysfunction, arrhythmogenic cardiomyopathy, and dual organ listings.

## 4 Discussion

Exception requests have always been a part of pediatric heart transplant listings, but the utility and impact have changed based on changes in the allocation system. In the current allocation system, exception requests are common with a high approval rate. Previous studies have demonstrated that more pediatric candidates are being listed with both 1A and 1B status exceptions under the current allocation system compared to previous policies [2]. During the study period, 35% (388/1108) of pediatric candidates were listed by exception.

The goal of creating the NHRB for pediatrics was to allow the experts that care for pediatric heart transplant candidates to decide if an exception request should be approved or denied, in addition to having a guidance document to provide indications for whom an exception request may be reasonable. Per the guidance document, the narratives submitted on behalf of candidates for an exception request are expected to demonstrate “both the medical urgency and potential for benefit comparable to that of other candidates” at the same status [5]. With this guidance, nearly all requests that followed the guidance were approved. This study demonstrates the NHRB has followed the OPTN guidance, while still having the opportunity to review candidates for exception requests that are outside the scope of the guidance document that may benefit from a higher listing status. Wright et al. recently published a study which demonstrated that center variation has been reduced, specifically for 1A exceptions since implementation of the NHRB [6]. However, there continues to be evidence of regional variability for 1B exceptions [6].

Since the allocation change in 2016, 81% of candidates were status 1A at the time of transplant [7]. This study shows that most of the status 1A exceptions being granted follow OPTN guidance. There has been an increase in exception requests for candidates with dilated, hypertrophic and restrictive cardiomyopathy, and an increased waitlist mortality for patients with hypertrophic and restrictive cardiomyopathy but not for patients with dilated cardiomyopathy who did not utilize a 1A exception [3]. The current policy resulted in a change in status from 1A to 1B for candidates with cardiomyopathy on inotropic support, and it is those candidates that now make up the majority of 1A exception requests. Candidates with DCM who are small, less than 10 kg, or have absolute or relative contraindications for a ventricular assist device (VAD) have been able to benefit from this guidance to obtain a 1A status. While overall VAD outcomes have improved, an exception request may still be reasonable for select candidates for which a VAD may be considered too high risk for that candidate or center. Examples of contraindications for VAD as described in the OPTN guidance document included candidates with severe gastrointestinal bleeding, hypercoagulable disorders, and other more subjective relative contraindications based on center experience [5].

Although the greatest number of requests for 1B exceptions were for 1V CHD, which is the focus of the guidance document for this status, many exceptions outside the guidance documents are sought and granted. While many exception requests were submitted for patients with failing Fontan physiology, a large portion were also submitted for 1V CHD patients with Glenn physiology deemed not Fontan candidates but were listed outpatient 1B by exception. The high approval may come from the Category 3 guidance statement “it appears appropriate to consider more urgent listing for many patients with single ventricle congenital heart disease, even where not supported by inotropes as an inpatient.” Adult candidates with single ventricle congenital heart disease are listed as adult status 4, which is equivalent to pediatric status 1B and thus the greatest number of requests for 1B exceptions were for 1V CHD. The guidance document for candidates with hypertrophic and restrictive cardiomyopathy only addresses criteria for 1A exceptions, not 1B, but 1B exception requests were often sought and granted. This study suggests that the addition of more 1B guidance may yield more consistent adherence from the review board, acknowledging that candidates with a 1B status still exhibit longer waitlist times and are less likely to be transplanted than patients listed at status 1A.

Candidates with coronary allograft vasculopathy requiring retransplantation have been shown to have a higher waitlist mortality than first‐time transplant candidates [8]. All 1A exception requests for candidates with CAV adhered to the guidance; however, requests for 1B exceptions, which recommend coronary revascularization, either transcatheter or surgical, were most often not followed.

Our analysis suggests that certain patient populations that follow OPTN guidance for exemption and almost always are approved should be included in the standard guidelines and not require exemption. For example, a 5 kg infant with dilated cardiomyopathy on inotropes, or 1V CHD candidates with failing Fontan physiology. Then, the use of exemptions would truly be for extenuating circumstances in which an institution believes the candidate should be a higher listing status than per standard guidelines. This study revealed that 38% of candidates during the study period had exemption requests submitted. With the high approval rate of 91%, 35% of candidates during the study period were listed by exception. If standard guidelines were more inclusive and granular, then perhaps there would be less of a need for exemption requests.

The current 3‐tiered status system makes it challenging to stratify and prioritize such a diverse population. The new anticipated continuous distribution system will allocate points to candidates based on individual medical and logistical criteria and thus eliminate the current 3‐tiered status system. The goal of continuous distribution is to make the allocation of organs more fair, flexible and equitable. However, it is unclear how a new continuous distribution system of hearts may impact the need and utilization of exception requests in the future.

Limitations to this study included the critical objective review of the request narrative and determining if the request followed the guidance document. Since 11% of the requests were outside the scope of these guidance, they were deemed not to be following the guidance but may have been appropriate requests. Regardless, there was still a high approval rate for the candidates that did not have a diagnosis outlined in the guidance. Additionally, this study did not analyze the mortality rates of those listed by exception compared to candidates listed by standard criteria as this has already been studied by Magnetta and colleagues [3].

In conclusion, most status 1A exception requests being approved follow OPTN guidance. While the greatest number of requests for 1B exceptions were for 1 V CHD, many exceptions outside the guidance are being sought and granted. Knowing the patterns of these exception requests may help inform future changes in priority status creation, possibly leading to more granular criteria similar to adult heart transplant criteria or aid in the creation of the new continuous distribution system.

## Data Availability

Research data are not shared.
